# Muscle mitochondrial energetics predicts mobility decline in well‐functioning older adults: The baltimore longitudinal study of aging

**DOI:** 10.1111/acel.13552

**Published:** 2022-01-20

**Authors:** Qu Tian, Brendan A. Mitchell, Marta Zampino, Kenneth W. Fishbein, Richard G. Spencer, Luigi Ferrucci

**Affiliations:** ^1^ Translational Gerontology Branch National Institute on Aging National Institutes of Health Baltimore Maryland USA; ^2^ Laboratory of Clinical Investigation National Institute on Aging National Institutes of Health Baltimore Maryland USA

**Keywords:** magnetic resonance spectroscopy, mitochondrial energetics, mobility decline, skeletal muscle, walking speed

## Abstract

**Background:**

Muscle mitochondrial dysfunction is associated with poor mobility in aging. Whether mitochondrial dysfunction predicts subsequent mobility decline is unknown.

**Methods:**

We examined 380 cognitively normal participants aged 60 and older (53%women, 22%Black) who were well‐functioning (gait speed ≥ 1.0 m/s) and free of Parkinson's disease and stroke at baseline and had data on baseline skeletal muscle oxidative capacity and one or more mobility assessments during an average 2.5 years. Muscle oxidative capacity was measured by phosphorus magnetic resonance spectroscopy as the post‐exercise recovery rate of phosphocreatine (k_PCr_). Mobility was measured by four walking tests. Associations of baseline k_PCr_ with mobility changes were examined using linear mixed‐effects models, adjusted for covariates. In a subset, we examined whether changes in muscle strength and mass affected these associations by adjusting for longitudinal muscle strength, lean mass, and fat mass.

**Results:**

Lower baseline k_PCr_ was associated with greater decline in all four mobility measures (β, *p*‐value: (0.036, 0.020) 6‐m usual gait speed; (0.029, 0.038) 2.5‐min usual gait speed; (0.034, 0.011) 6‐m rapid gait speed; (−0.042, <0.001) 400‐m time). In the subset, further adjustment for longitudinal muscle strength, lean mass, and fat mass attenuated longitudinal associations with changes in mobility (Δβ reduced 26–63%).

**Conclusion:**

Among initially well‐functioning older adults, worse muscle mitochondrial function predicts mobility decline, and part of this longitudinal association is explained by decline in muscle strength and mass. Our findings suggest that worse mitochondrial function contributes to mobility decline with aging. These findings need to be verified in studies correlating longitudinal changes in mitochondrial function, muscle, and mobility performance.

## Graphical abstract

Those with low baseline muscle mitochondrial function had greater decline in usual gait speed and greater increase in 400 m walk time than those with high baseline function.

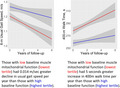

## 1 INTRODUCTION

Slow gait and mobility decline are common in older age and are associated with adverse outcomes such as mobility disability, reduced quality of life, loss of autonomy in daily life activities, and mortality (Cooper et al., 2010; Guralnik et al., 1995; Perera et al., 2016). Such a decline may arise from impairments in the central nervous system (CNS), musculoskeletal system, and metabolic systems (Ferrucci et al., 2000, 2016). Previous findings suggest that age‐related decline of mitochondrial function may contribute to loss of mobility. Compared to young adults, older adults have lower skeletal muscle oxidative capacity (Conley et al., 2000) and higher metabolic cost of walking (Das Gupta et al., 2019; Mian et al., 2006). In older age, decreased muscle oxidative capacity and increased metabolic cost of walking are associated with poorer mobility performance (Coen et al., 2013; Schrack et al., 2013).

Proposed mechanisms underlying the relationship between mitochondrial dysfunction and mobility decline include impairments in energy production and energy utilization. Energy production can be impaired due to age‐related decline of mitochondrial function, possibly through a combination of lack of energy, increased oxidative stress, oxidative damage to mitochondrial DNA and the complexes of the electron transport chain, and altered gene expression (Gonzalez‐Freire et al., 2015; Peterson et al., 2012). Energy utilization may also be impaired with aging, particularly through the molecular damage and anatomical damage secondary to oxidative stress and other age‐related processes that may interfere with function. The high energetic demands of skeletal muscle render it particularly vulnerable to reactive oxygen species‐related damage (Jackson & O'Farrell, 1993). Therefore, a subtle decline of mitochondrial function may substantially affect skeletal muscle function and lead to subsequent mobility decline (Barclay, 2017). Indeed, recent studies have shown that poorer skeletal muscle mitochondrial function, measured as oxidative capacity, is cross‐sectionally associated with poorer mobility performance in older adults and that this relationship is mediated by skeletal muscle strength (Choi et al., 2016; Zane et al., 2017). However, whether skeletal muscle mitochondrial function predicts subsequent mobility decline has not been investigated.

A powerful non‐invasive approach to assess skeletal muscle mitochondrial function in vivo is to measure the post‐exercise rate of recovery of phosphocreatine (k_PCr_) using phosphorus magnetic resonance spectroscopy (^31^P‐MRS) (Conley et al., 2000; McCully et al., 1991, 1993). k_PCr_ provides an estimate of in vivo skeletal muscle oxidative capacity, a metric for mitochondrial function, and this interpretation is further supported by its association with the citrate synthase activity of muscle (McCully et al., 1993) and with mitochondrial respiration measured in biopsy tissue (Gonzalez‐Freire et al., 2018). Further, unlike VO_2_max obtained during treadmill testing, k_PCr_ assessment is not affected by postural or balance impairments.

The primary goal of this study was to examine whether skeletal muscle mitochondrial function, measured as k_PCr_, would predict subsequent mobility changes in a sample of initially well‐functioning older adults. We hypothesized that lower k_PCr_ at baseline would be associated with greater mobility decline over time. We also hypothesized that this longitudinal association would be mediated at least in part by changes in lower extremity muscle strength and mass. Understanding these longitudinal relationships can provide new insights into mechanisms underlying mobility decline and help design early prevention and intervention strategies.

## 2 RESULTS

Baseline participant characteristics are presented in Table 1. The majority of the participants had repeated measures of mobility (73%). Participant characteristics were similar between the overall sample and those with repeated mobility assessments (Table 1). In both samples, lower k_PCr_ was associated with older age, higher body mass index, and lower muscle strength. Women had lower k_PCr_ than men. Blacks had lower k_PCr_ than whites.

**TABLE 1:** Baseline participant characteristics

	Overall sample (*n* = 380)			Subset with repeated mobility (*n* = 279)		
	Mean (SD) or N (%)	Range	Correlations with k_PCr_, *p*‐value	Mean (SD) or N (%)	Range	Correlations with k_PCr_, *p*‐value
Demographics						
Age, years	72.9 (7.8)	60–91	**<0.001**	73.9 (7.7)	60–91	**0.022**
Women	203 (53)	–	**<0.001**	149 (53)	–	**<0.001**
Blacks	83 (22)	–	**<0.001**	53 (19)	–	**<0.001**
Height, cm	168 (9)	147–192	0.380	168 (9)	148–192	0.931
Body mass index, kg/m^2^	26.7 (4.2)	17–43	**0.047**	26.3 (4.1)	17–38	**0.020**
k_PCr_, s^−1^	0.0207 (0.0049)	0.0114–0.0404	–	0.0202 (0.0046)	0.0114–0.0404	–
PCr depletion, %	55 (11)	33–91	0.727	55 (11)	33–91	0.302
Muscle strength, N·m	111 (38) (*n* = 334)	34–248 (*n* = 334)	**0.015** (*n* = 334)	108 (37) (*n* = 245)	34–244 (*n* = 245)	0.085 (*n* = 245)
Total fat mass, kg	26 (9.6) (*n* = 365)	5.3–65 (*n* = 365)	**0.006** (*n* = 365)	25.3 (9.3) (*n* = 273)	5.3–55 (*n* = 273)	**0.002** (*n* = 273)
Total lean mass, kg	46 (9.6) (*n* = 365)	31–72 (*n* = 365)	0.352 (*n* = 365)	46 (9.7) (*n* = 273)	31–71 (*n* = 273)	0.745 (*n* = 273)
Mobility assessment						
6‐m usual gait speed, m/s	1.26 (0.17)	1.00–1.81	**<0.001**	1.25 (0.16)	1.00–1.81	**<0.001**
2.5‐min usual gait speed, m/s	1.26 (0.14)	0.88–1.64	**<0.001**	1.26 (0.13)	0.93–1.61	**<0.001**
6‐m rapid gait speed, m/s	1.88 (0.30)	1.32–3.21	**0.002**	1.87 (0.31)	1.32–3.21	**0.049**
400‐m walk time, sec	265 (38) (*n* = 374)	162–418 (*n* = 374)	**<0.001** (*n* = 374)	265 (36) (*n* = 267)	162–419 (*n* = 267)	**<0.001** (*n* = 267)
Follow‐up time, years	2.5 (1.8)	0–6	–	3.5 (1.2)	1–6	–
Number of visits per participant	2.4 (1.2)	1–7	–	2.9 (1.0)	2–7	–

Note

*p*‐Values were based on Pearson's coefficient for continuous variables and independent t tests for binary variables. Bold numbers reflect significant correlations at two‐sided *p *< 0.05.

Cross‐sectionally, lower k_PCr_ was significantly associated with slower 6‐m usual gait speed and 2.5‐min usual gait speed, and longer 400‐m walk time at baseline after covariate adjustment (Table 2). The association between k_PCr_ and 6‐m rapid gait speed was not statistically significant (Table 2). These cross‐sectional associations remained similar in the subset of participants who had data on thigh muscle strength, total lean mass, and total fat mass (Table S1, Model 1). Adjustment for muscle strength, lean mass, and fat mass attenuated cross‐sectional associations except for the association with 400‐m walk time (Table S1, Model 4).

**TABLE 2:** Associations between k_PCr_ and mobility (*n* = 380)

	Cross‐sectional associations between k_PCr_ and mobility at baseline		Longitudinal associations between baseline k_PCr_ and subsequent mobility changes	
	β (95% CI)	*p*‐Value	β (95% CI)	*p*‐Value
6‐m usual gait speed				
Age	−0.039 (−0.050, −0.023)	**<0.001**	−0.006 (−0.009, −0.002)	**0.004**
k_PCr_	0.111 (0.010, 0.211)	**0.031**	0.036 (0.006, 0.067)	**0.020**
2.5‐min usual gait speed				
Age	−0.042 (−0.055, −0.029)	**<0.001**	−0.004 (−0.008, −0.001)	**0.013**
k_PCr_	0.143 (0.045, 0.241)	**0.005**	0.029 (0.002, 0.056)	**0.038**
6‐m rapid gait speed				
Age	−0.058 (−0.071, −0.046)	**<0.001**	−0.002 (−0.005, 0.002)	0.283
k_PCr_	0.040 (−0.052, 0.131)	0.397	0.034 (0.008, 0.060)	**0.011**
400‐m walk time (*n* = 374)				
Age	0.069 (0.057, 0.082)	**<0.001**	0.009 (0.005, 0.012)	**<0.001**
k_PCr_	−0.142 (−0.238, −0.045)	**0.004**	−0.042 (−0.067, −0.018)	**<0.001**

Note

Models were adjusted for baseline age, sex, extent of PCr depletion during exercise, and body mass index over time. Values of mobility measures and k_PCr_ were computed as standardized *Z* scores based on mean and standard deviation at baseline. Bold numbers reflect significant associations at two‐sided *p* < 0.05.

**FIGURE 1:**
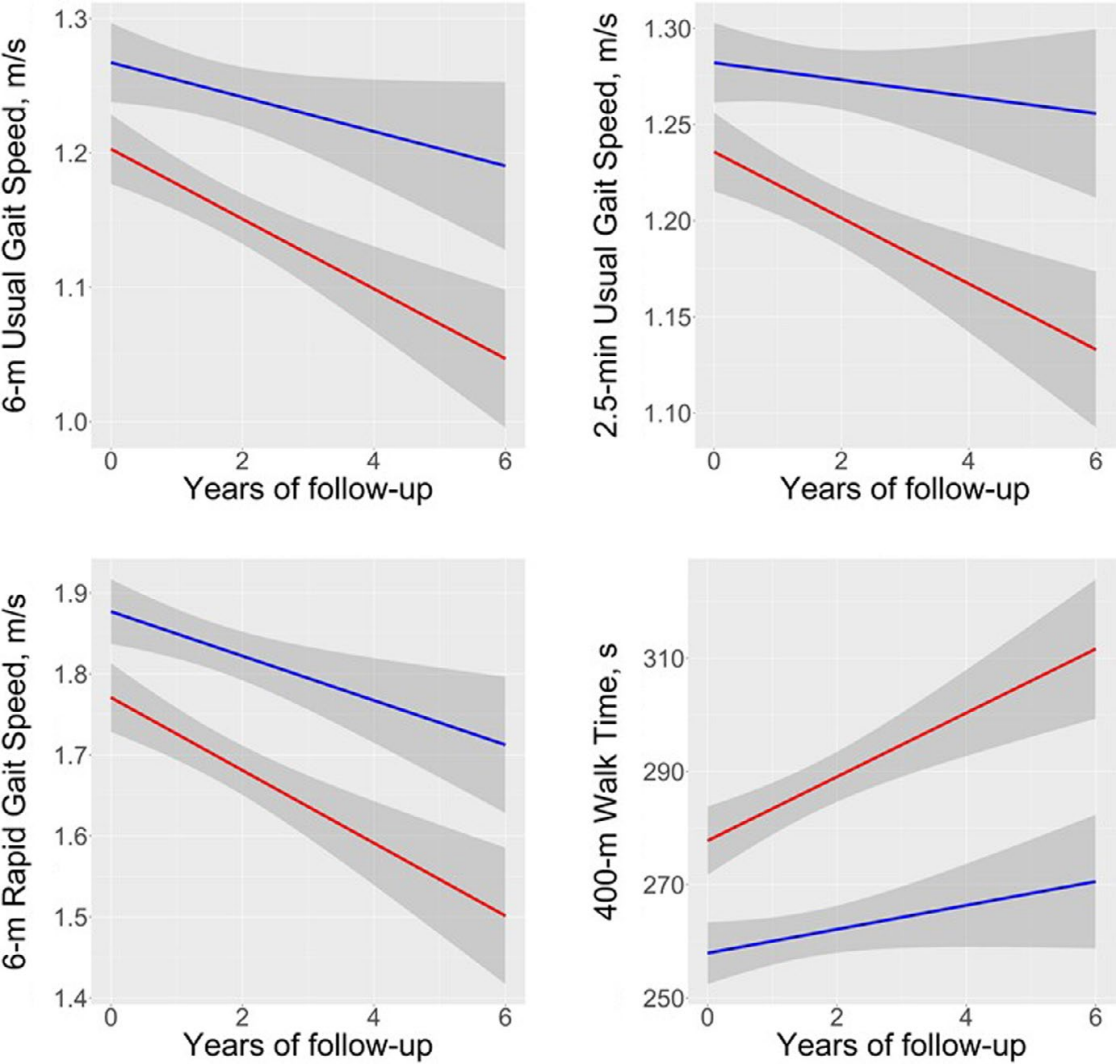
Predicted mobility changes among those with low (lowest tertile: red) and high (highest tertile: blue) baseline k_PCr_. Legend: Predicted mobility changes are adjusted for covariates, including baseline age, sex, extent of PCr depletion during exercise, and body mass index over time

Longitudinally, lower baseline k_PCr_ was significantly associated with greater decline in all four walking performance measures after covariate adjustment (Table 2). Figure 1 showed predicted mobility changes over time among participants in the highest and lowest tertiles of baseline k_PCr_. These longitudinal associations did not substantially change when analyzed in the subset of participants who had data on thigh muscle strength, total lean mass, and total fat mass (Table S2, Model 1). Adjustment for longitudinal muscle strength substantially attenuated longitudinal associations between baseline k_PCr_ and changes in all four walking performance measures (Δβ reduced 18%–28% (Table S2, Model 2). Figure S1 showed predicted mobility changes over time among participants in the highest and lowest tertiles of baseline k_PCr_ after adjustment for longitudinal muscle strength. Additional adjustment for longitudinal total lean mass and fat mass further attenuated these longitudinal associations (Δβ reduced 26% to 63% (Table S2, Model 4).

## 3 DISCUSSION

This study established two important findings. First, in this sample of initially well‐functioning community‐dwelling older adults, lower skeletal muscle mitochondrial oxidative capacity predicts subsequent mobility decline. Second, longitudinal associations of baseline k_PCr_ with subsequent mobility changes are substantially attenuated after accounting for longitudinal thigh muscle strength, total lean mass, and total fat mass.

Our study extended prior cross‐sectional research by examining longitudinal changes in mobility, as well as by focusing on initially well‐functioning older adults, and by examining the effect of longitudinal change in muscle strength. We found that that the post‐exercise rate of recovery of phosphocreatine was a strong predictor of mobility decline measured by four different walking tests. Compared to those with higher baseline kPCr (i.e., highest tertile), those with lower baseline kPCr (i.e., lowest tertile) had 0.05 m/s greater decline in usual gait speed and 18 s greater increase in 400‐m walk time over 3.5 years, which were considered small but clinically meaningful change (Perera et al., 2014). Clinical implications of our results include the concept that older adults with poor mitochondrial function may represent a target group for early intervention to maintain functional independence with aging. Pharmacological or nonpharmacological strategies aimed at improving muscle function may slow down the decline of mobility with aging.

The longitudinal relationship between skeletal muscle mitochondrial function and mobility decline appeared to be mediated by the change in thigh muscle strength, lean mass, and fat mass. Adjustment for longitudinal muscle strength resulted in a relative reduction in the regression coefficient of ~20–30%, and further adjustment for total lean mass and fat mass led to a reduction in the coefficient of ~30–60%. This finding suggests that muscle strength and mass may have a complementary mediating effect on the relationship between skeletal muscle oxidative capacity and change in mobility. Low mitochondrial function may negatively affect skeletal muscle function and in turn affect mobility. We note that the longitudinal associations between baseline mitochondrial function and change in 400‐m walk time remained statistically significant after muscle strength adjustment. This finding may suggest that performance on a challenging mobility task is directly dependent on the ability of mitochondria to sustain energy production over an extended period of time, although neurological control may also play a role. Indeed, recent data have suggested a strong link between mitochondria and the central nervous system (Picard & McEwen, 2014). Our group has also shown that 400‐m walk time is a strong predictor of future cognition and brain structure (Tian et al., 2017, 2019). Overall, central nervous system‐related mechanisms underlying mitochondrial function and performance on a challenging mobility task warrant further investigations.

Our observation of cross‐sectional associations between muscle mitochondrial oxidative capacity and mobility, especially the strong association with 400‐m walk time, a challenging mobility measurement, is consistent with previous results (Choi et al., 2016; Coen et al., 2013; Zane et al., 2017). However, in contrast to those studies, we did not find a significant cross‐sectional association with 6‐m rapid gait speed. This finding could be due to differences in sample characteristics between the previous and current studies, although both studies used BLSA participants’ data. Study samples in previous studies were characterized by a wide range of both age and gait speed, while the present study selected well‐functioning participants with baseline gait speed equal to or >1.0 m/s. The present study also focused on cognitively normal participants who were free of PD and stroke at baseline to minimize the influence of neurological conditions on the relationships between skeletal muscle mitochondrial function and mobility changes over time (Busse et al., 2006).

Among the strengths of our study is its comprehensive examination of four different mobility performance measures, including usual and rapid pace walking tests, short‐ and long‐distance walking tests. This allowed us to differentially examine which mobility changes were more strongly predicted by muscle mitochondrial function. In addition, vigorous adjudication of cognitive impairment and assessment of PD and stroke allowed us to focus on participants without overt neurological conditions to better understand how skeletal muscle mitochondrial function contributes to mobility decline. Further, we focused on initially well‐functioning older adults, which allowed us to investigate mobility change in a more homogenous sample as an inception cohort.

Although our study examined a relatively large sample from a well‐characterized cohort and applied advanced methods to establish the longitudinal relationship between mitochondrial function and mobility change, it nevertheless has certain limitations. First, the BLSA population is healthier and better educated than the general population, so these results may not be entirely generalizable. However, we believe that it is highly likely that our results, based on fundamental physiological parameters, would apply more broadly. Second, thigh muscle strength data were available for only a subset of participants, leading to somewhat reduced statistical power. Nevertheless, we were still able to study a large sample of 334 participants for whom thigh muscle strength data were available. In addition, our analyses showed that both cross‐sectional and longitudinal associations in the subset were similar to those in the overall sample.

Overall, our results suggest that skeletal muscle mitochondrial function predicts subsequent mobility decline. This relationship may be explained by a limitation of energy provided by muscle mitochondria, which negatively affects the production of muscle strength. These findings suggest that strategies to prevent the decline of mitochondrial function with aging and enhance mitochondrial function applied at an early stage may prevent or slow down the mobility decline with aging.

## 4 EXPERIMENTAL PROCEDURES

### 4.1 Study population

Participants were drawn from the Baltimore Longitudinal Study of Aging (BLSA), a prospective study with a continuous enrollment since 1958 (Ferrucci, 2008; Shock et al., 1984). In brief, participants must be free of major chronic conditions and cognitive impairment at the time of enrollment. Follow‐up visits then occur at different intervals depending on a participant's age (e.g., every 2 years for ages 60–79 and every year for ages 80 and older). Diagnoses of cognitive impairment and dementia followed standard BLSA procedures, described previously (Driscoll et al., 2009). In BLSA, diagnoses of dementia and Alzheimer's disease have continued to follow, respectively, the Diagnostic and Statistical Manual, third edition, revised (DSM‐III‐R) (American Psychiatric Association, 1987) and the National Institute of Neurological and Communication Disorders and Stroke‐Alzheimer's Disease and Related Disorders Association criteria (McKhann et al., 1984). Mild cognitive impairment was determined using the Petersen criteria (Petersen et al., 1999). Diagnoses of Parkinson's disease (PD) and stroke were assessed during the follow‐up visits by nurse practitioners based on the information provided by the medical history, treatments, and diagnostic tests. The National Institutes of Health approved the BLSA protocol. All participants provided written informed consent at each visit.

In this study, data were collected between April 2013 and December 2019. The first concurrent assessment of mitochondrial function and mobility was considered as baseline. We identified 380 cognitively normal participants aged 60 and older (53% women, 22% Black) who were well‐functioning (gait speed ≥1.0 m/s) and free of Parkinson's disease and stroke at baseline, and who had baseline k_PCr_ measurements as well as one or more measures of mobility over an average of 2.5 (SD = 1.8) years. In this study, the average number of visits was 2.4 (SD = 1.2) per participant.

### 4.2 Skeletal muscle oxidative capacity determined by 31P MRS

In vivo ^31^P‐MRS measurements of phosphorus‐containing metabolites were obtained from the quadriceps muscles using a 3T Achieva MR scanner (Philips, Best, The Netherlands), as described previously (Choi et al., 2016; Zane et al., 2017).

Participants were positioned supine in the bore of the scanner with a foam wedge underneath the knee to maintain slight flexion (30°), with thighs and hips secured with straps to reduce displacement during exercise. As instructed, participants performed a fast, intense, ballistic knee extension exercise designed to deplete PCr in the quadriceps muscles with minimal acidification, permitting assessment of maximal oxidative phosphorylation (Coen et al., 2013).

A series of pulse‐acquire ^31^P‐MRS spectra were obtained before, during, and after exercise using a 10‐cm ^31^P‐tuned, flat surface coil (PulseTeq, Surrey, United Kingdom) that was secured over the vastus lateralis muscle of the left thigh. ^31^P nuclei were excited with 90° adiabatic radio frequency (RF) pulses with an inter‐pulse delay time TR = 1.5 s, a four‐step phase cycle, and four averages, resulting in a temporal resolution of 6 s. A total of 75 spectra were obtained over the 60 s before, 30 s during, and 360 s after exercise; the total duration of MR data acquisition was 7.5 min (Choi et al., 2016).

Spectra were processed using jMRUI (version 5.2) and fit in the time domain using a nonlinear least‐squares algorithm (AMARES) (Naressi et al., 2001; Vanhamme et al., 1999). Maximum muscular oxidative capacity was characterized by the post‐exercise PCr recovery rate constant, k_PCr_, which was determined by fitting time‐dependent changes in PCr peak area using the following mono‐exponential function:

$$\text{PCr}\left(t\right)={\text{PCr}}_{0}+\Delta \text{PCr}\times \left(1‐e^{‐t/\tau }\right),$$

where PCr_0_ was the PCr signal amplitude at the end of the exercise, that is, the beginning of the recovery, ΔPCr was the decrease in PCr observed from baseline to the end of the exercise, τ was the PCr recovery time constant, and k_PCr_ was the PCr recovery rate constant determined as 1/τ (Choi et al., 2016; Zane et al., 2017). For quality control purposes, data were accepted only from experiments in which PCr was depleted by at least 33% during ballistic exercise.

### 4.3 Mobility assessment

Mobility performance was measured using four walking tests, including usual and rapid gait speed over 6 m, usual gait speed over 2.5 min, and 400‐m walk time. For usual and rapid gait speed over 6 m, two trials were recorded, and the faster trial was used for analysis. 2.5‐min usual gait and 400‐m walk time were measured during the long distance corridor walk (Simonsick et al., 2001). Participants were instructed to walk 2.5‐min at their usual pace as a warm‐up and then to walk 400 m as fast as they could. 400‐m walk task is considered a challenging mobility task.

### 4.4 Muscle strength and mass

Maximal quadriceps muscle strength was measured using an isokinetic dynamometer (Biodex Multi‐Joint System‐Pro with Advantage Software V.4X, Biodex Medical Systems, Inc., Shirley, NY, USA) (Hartmann et al., 2009). Maximum quadriceps muscle strength was defined as the greatest of three consecutive values of torque (Nm) measured by the force generated from left leg concentric knee extensor contraction at an angular velocity of 30° per second (Hartmann et al., 2009). Total body dual‐energy X‐ray absorptiometry (DEXA) was performed using a Prodigy Scanner (General Electric, Madison, WI) and analyzed with version 10.51.006 software. DEXA uses tissue absorption of X‐ray beams to identify lean body mass and fat mass with quantitative data (Van Loan & Mayclin, 1992).

### 4.5 Statistical analysis

Bivariate correlations of participant characteristics with baseline k_PCr_ were examined using Pearson correlation coefficients for continuous variables and t tests for binary variables.

We first examined associations between baseline k_PCr_ and subsequent mobility measures using linear mixed‐effects models. Models were adjusted for baseline age, sex, extent of PCr depletion, and body mass index (BMI) over time. Fixed effects included interval (follow‐up time in years), k_PCr_, k_PCr_ × interval, age, age × interval, sex, sex × interval, BMI, BMI × interval, depletion, depletion × interval. Random effects included the intercept and interval.

We then examined the hypothesis that longitudinal associations between baseline mitochondrial function and subsequent mobility decline were mediated by changes in thigh muscle strength and mass in a subset of participants. Specifically, we adjusted longitudinal data on thigh muscle strength, total lean mass, and total fat mass in addition to other covariates listed above. All analyses were performed using RStudio version 4.0.2 (Boston, MA). Significance was set at two‐sided *p* < 0.05.

## CONFLICT OF INTEREST

The authors have no conflict of interest to declare.

## AUTHOR CONTRIBUTIONS

QT designed the study, developed the statistical analysis plan, and drafted the manuscript. BAM performed statistical analyses and drafted the manuscript. ZM, KWF, and RGS critically evaluated the manuscript and edited the manuscript. LF designed the study and edited the manuscript. All authors approved the manuscript.

## Supporting information

Fig S1

Table S1‐S2

## Data Availability

Available upon request.
