# Supplementary figures and images for: Muscle mitochondrial energetics predicts mobility decline in well‐functioning older adults: The baltimore longitudinal study of aging
Source: Aging Cell. 2022 Jan 20;21(2):e13552. doi: 10.1111/acel.13552 (PMC8844110; doi:10.1111/acel.13552)

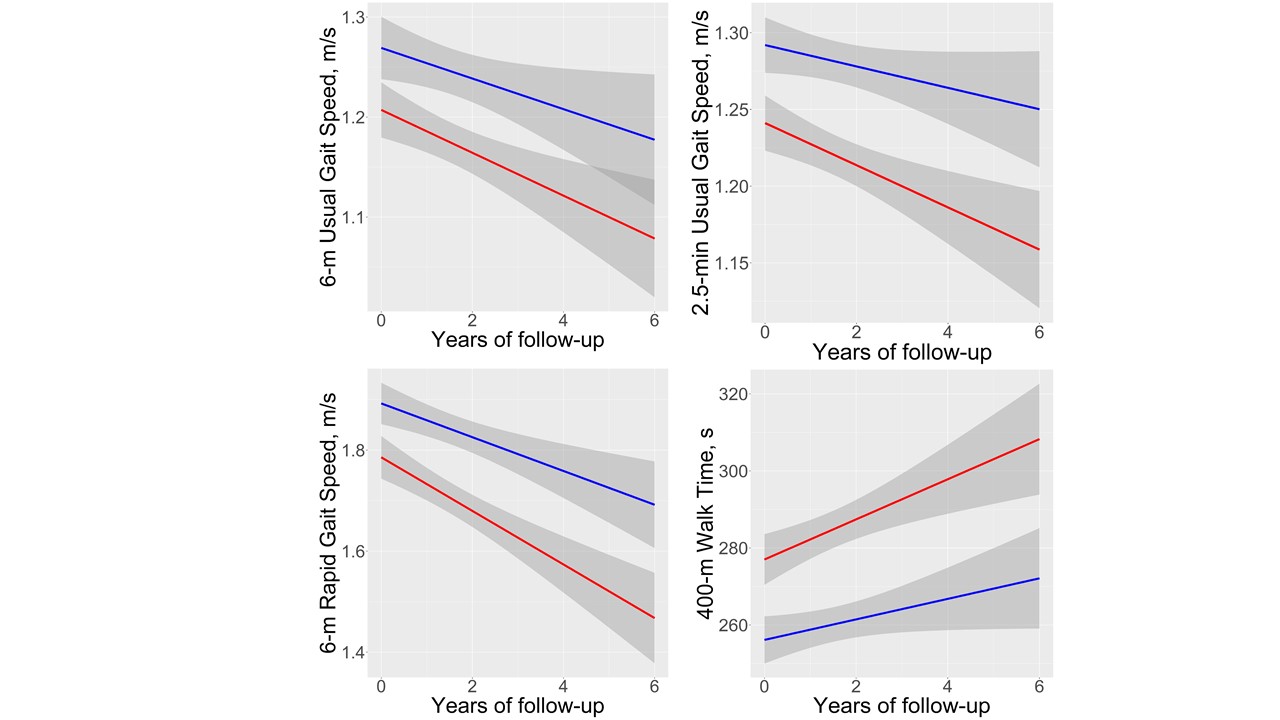

Supplement: Supplementary file 1 — Fig S1 [file ACEL-21-e13552-s002.jpg]
