# Supplementary material for: Muscle mitochondrial energetics predicts mobility decline in well‐functioning older adults: The baltimore longitudinal study of aging
Source: Aging Cell. 2022 Jan 20;21(2):e13552. doi: 10.1111/acel.13552 (PMC8844110; doi:10.1111/acel.13552)
Supplement: Supplementary file 2 — Table S1‐S2 [file ACEL-21-e13552-s001.docx]

**Table S1. Cross-sectional associations between baseline k_PCr_ and mobility performance with adjustment for baseline thigh muscle strength, total lean mass, and total fat mass**

|  | **Model 1: covariates** | **Model 2:**  **Model 1 + muscle strength** | **Model 3:**  **Model 1 +**  **fat mass +**  **lean mass** | **Model 4:**  **Model 1 + muscle strength + fat mass + lean mass** |
| --- | --- | --- | --- | --- |
|  | β (95% CI)  p-value | | | |
| **6-m usual gait speed (n=334)** | 0.089  (-0.017, 0.195) 0.098 | 0.080  (-0.025, 0.186) 0.136 | 0.074  (-0.033, 0.180)  0.174  (n=321) | 0.070  (-0.037, 0.177)  0.198  (n=321) |
| **2.5-min usual gait speed (n=334)** | 0.127  (0.023, 0.232) **0.017** | 0.113  (0.011, 0.215) **0.031** | 0.121  (0.018, 0.225)  **0.022**  (n=321) | 0.111  (0.007, 0.214)  **0.036**  (n=321) |
| **6-m rapid gait speed (n=334)** | 0.030  (-0.065, 0.126) 0.534 | 0.027  (-0.069, 0.123) 0.581 | 0.009  (-0.086, 0.104)  0.850  (n=321) | 0.008  (-0.088, 0.103)  0.878  (n=321) |
| **400-m walk time (n=331)** | -0.126  (-0.228, -0.023) **0.016** | -0.129  (-0.221, -0.037) **0.006** | -0.116  (-0.209, -0.024)  **0.014**  (n=319) | -0.112  (-0.202, -0.022)  **0.015**  (n=319) |

Note. Covariates included baseline age, sex, extent of PCr depletion during exercise, and body mass index. Values of mobility measures, k_PCr_, muscle strength, fat mass, and lean mass were computed as standardized Z scores based on mean and standard deviation at baseline. Bold numbers reflect significant associations at two-sided p<0.05.

**Table S2. Longitudinal associations between baseline k_PCr_ and mobility changes with adjustment for longitudinal thigh muscle strength, total lean mass, and total fat mass**

|  | **Model 1: covariates** | **Model 2:**  **Model 1 + muscle strength** | **Model 3:**  **Model 1 +**  **fat mass +**  **lean mass** | **Model 4:**  **Model 1 + muscle strength + fat mass + lean mass** |
| --- | --- | --- | --- | --- |
|  | **β (95% CI)**  **p-value** | | | |
| **6-m usual gait speed (n=334)** | 0.040  (0.008, 0.073)  **0.015** | 0.029  (-0.005, 0.063) 0.093 | 0.037  (0.002, 0.071)  **0.039**  (n=321) | 0.021  (-0.016, 0.058)  0.271  (n=321) |
| **2.5-min usual gait speed (n=334)** | 0.026  (-0.004, 0.055) 0.086 | 0.021  (-0.010, 0.052) 0.174 | 0.014  (-0.017, 0.046)  0.371  (n=321) | 0.011  (-0.023, 0.044)  0.535  (n=321) |
| **6-m rapid gait speed (n=334)** | 0.038  (0.010, 0.066) **0.007** | 0.031  (0.001, 0.060) **0.043** | 0.034  (0.005, 0.063)  **0.022**  (n=321) | 0.028  (-0.004, 0.059)  0.086  (n=321) |
| **400-m walk time (n=331)** | -0.046  (-0.072, -0.019) **<0.001** | -0.033  (-0.056, -0.011)  **0.004** | -0.024  (-0.046, -0.002)  **0.035**  (n=319) | -0.017  (-0.041, 0.006)  0.153  (n=319) |

Note: Same as Table S1.

**Figure S1. Predicted mobility changes with further adjustment for longitudinal muscle strength among those with low (lowest tertile: red) and high (highest tertile: blue) baseline k_PCr._** Legend: Covariates included baseline age, sex, extent of PCr depletion, and body mass index over time.
